# Safety and mortality outcomes for direct oral anticoagulants in renal transplant recipients

**DOI:** 10.1371/journal.pone.0285412

**Published:** 2023-05-16

**Authors:** Christine Firth, Fadi Shamoun, Michael Apolinario, Elisabeth S. Lim, Nan Zhang, Mira T. Keddis

**Affiliations:** 1 Department of Cardiovascular Diseases, Scottsdale, AZ, United States of America; 2 Department of Internal Medicine, Scottsdale, AZ, United States of America; 3 Department of Quantitative Health Sciences, Scottsdale, AZ, United States of America; 4 Department of Nephrology, Mayo Clinic, Scottsdale, AZ, United States of America; Faculty of Medicine, Saint-Joseph University, LEBANON

## Abstract

**Purpose:**

Direct oral anticoagulants (DOACs) are increasingly used in renal transplant recipients (RTR), but relatively understudied in this population. We assess the safety of post-transplant anticoagulation with DOACs compared to warfarin.

**Methods:**

We conducted a retrospective study of RTRs at the Mayo Clinic sites (2011-present) that were anticoagulated for greater than 3 months excluding the 1^st^ month post-transplant. The main safety outcomes were bleeding and all-cause mortality. Concomitant antiplatelet and interacting drugs were noted. DOAC dose adjustment was assessed according to common US prescribing practices, guidelines, and/or FDA labeling.

**Results:**

The median follow-up was longer for RTRs on warfarin (1098 days [IQR 521, 1517]) than DOACs (449 days [IQR 338, 942]). Largely, there were no differences in baseline characteristics and comorbidities between RTRs on DOACs (n = 208; apixaban 91.3%, rivaroxaban 8.7%) versus warfarin (n = 320). There was no difference in post-transplant use of antiplatelets, immunosuppressants, most antifungals assessed, or amiodarone. There was no significant difference in incident major bleeding (8.4 vs. 5.3%, p = 0.89), GI bleeding (4.4% vs. 1.9%, p = 0.98), or intra-cranial hemorrhage (1.9% vs. 1.4%, p = 0.85) between warfarin and DOAC. There was no significant difference in mortality in the warfarin group compared to DOACs when adjusted for follow-up time (22.2% vs. 10.1%, p = 0.21). Rates of post-transplant venous thromboembolism, atrial fibrillation or stroke were similar between the two groups. 32% (n = 67) of patients on DOACs were dose reduced, where 51% of those reductions were warranted. 7% of patients that were not dose reduced should have been.

**Conclusions:**

DOACs did not have inferior bleeding or mortality outcomes compared to warfarin in RTRs. There was greater use of warfarin compared to DOACs and a high rate of improper DOAC dose reduction.

## Introduction

Venous thromboembolism (VTE) and atrial fibrillation are common in renal transplant recipients (RTR). There is a fairly high rate of incident atrial fibrillation both pre-transplant (6%) and post-transplant (7% within 1^st^ 3 years), which is associated with poorer post-transplant outcomes including graft failure, adverse cardiovascular events and mortality [1–3]. There is also a high rate of incident VTE in RTR compared to the general population (nearly 7 times higher), associated with higher mortality and graft loss compared to matched RTR that did not experience post-transplant VTE [4].

Anticoagulation in RTR is complicated by additional risks and challenges inherent to this population including increased bleeding and thrombosis, fluctuating renal function, and drug interactions [5–8]. Direct-acting oral anticoagulants (DOAC) are recommended over warfarin for anticoagulation in atrial fibrillation and VTE in the general population due to similar efficacy and similar or improved safety (superior bleeding profile, particularly intracerebral hemorrhage [ICH]) as well as ease of use [9, 10]. DOACs are increasingly used in RTR, but remain relatively understudied in this population [11]. Clinicians may be hesitant to use DOACs due to heightened concerns regarding the aforementioned variables in RTR compared to warfarin, particularly regarding the partial renal clearance of DOACs with the propensity for kidney injury in this setting [12]. Dosing concerns are exacerbated by the lack of adequate testing to ensure therapeutic treatment.

We aim to assess the safety profile of DOACs compared to warfarin and examine prescribing practices with respect to established dosing guidelines in a cohort of RTR.

## Materials and methods

The protocol for this study was approved by the Mayo Clinic institutional review board (IRB 20–013364). The requirement for informed consent was waived due to the retrospective nature of the study. We conducted a retrospective study of RTRs completed at the Mayo Clinic sites (January 01, 2011 through February 28, 2021) that were anticoagulated for greater than three months for any indication excluding the first month and within the first year post-transplant. We were interested in the anticoagulation effect in patients that were on a more prolonged duration of anticoagulation (e.g. atrial fibrillation, pulmonary embolism and/or VTE with decision for lifetime treatment). Those with prescriptions or medication data for greater than three months would likely have at least 6 months of therapy in typical clinical practice (i.e. excludes those that were treated for 3 months or less which would encompass indications such as provoked deep vein thrombosis or superficial venous thrombosis that required treatment). The first month was excluded in order to exclude any immediate post-transplant complications that may affect graft function and drug dosing. At our transplant center, RTR are evaluated multiple times per week during the first four weeks with longer intervals of follow-up after a one month period. The protocol beyond one month post-transplant includes visits at four, eight, and twelve months, then annually thereafter. Anticoagulants assessed included warfarin, apixaban, rivaroxaban, dabigatran, and edoxaban. DOAC doses were also recorded. Medication data was collected using the electronic medical record (EMR) for both the inpatient and outpatient settings, including administered medications, medication reconciliations and prescription data. Those with mechanical valves, antiphospholipid antibody syndrome, and ventricular thrombus were excluded.

Demographic data and cardiovascular risk factors were collected from the EMR at baseline (time of renal transplant) including age, gender, body mass index (BMI), history of diabetes mellitus, coronary artery disease (CAD), hypertension, peripheral arterial disease (PAD), congestive heart failure (CHF), atrial fibrillation, VTE, gastrointestinal (GI) bleed, ICH, ischemic stroke, or smoking. Variables were defined using ICD-9-CM and ICD-10-CM diagnosis codes (S8 Table). The following potential interacting medications were noted: amiodarone, fluconazole, itraconazole, voriconazole, posaconazole, diltiazem, tacrolimus, cyclosporine, or mammalian target of rapamycin inhibitor or belatacept. Concomitant anti-platelet medications were also recorded (aspirin, clopidogrel, prasugrel, or ticagrelor). The aforementioned relevant medications were recorded if noted any time during the first year post transplant. Creatinine (Cr) labs were collected (baseline, all Cr measurements within the first year post-transplant, and the value closest to last follow-up). All available INR labs were collected within the study period.

The main safety outcomes assessed comparing RTR on DOAC versus warfarin were bleeding (major bleeding, ICH, and GI bleeding) and all-cause mortality. Major bleeding was defined as ICH, GI bleeding involving hemorrhage, other forms of hemorrhage, and bleeding into critical sites such as ocular, pericardium, joint, etc (S8 Table). Incident VTE, ischemic stroke and renal graft failure were also noted stratified by anticoagulant group. Outcomes were defined using ICD-9-CM and ICD-10-CM diagnosis codes (S8 Table) and mortality data were obtained from the EMR. Random samples of the major bleeding outcome were manually verified through review of the EMR to ensure outcome accuracy (described in the supporting information). DOAC dose adjustment and correctness was assessed according to common US prescribing practices, guidelines, and/or FDA labeling (described in the supporting information).

### Statistical analysis

Demographic and outcome data collected from the EMR were compiled into an operational database within REDCap (Vanderbilt University, Nashville, TN). Descriptive statistics were used to summarize patient demographics and medical comorbidities to identify any differences between anticoagulant groups. Categorical variables were compared with the chi-square test and displayed as counts and percentages. Continuous variables were compared using Kruskal-Wallis test and expressed as means and standard deviations (SD) or median and interquartile range (IQR).

Overall mortality stratified by anticoagulation groups was evaluated using Kaplan-Meier curves and compared between the two groups using log-rank test. Other outcomes (bleeding outcomes, ICH, VTE, stroke and renal failure) cumulative incidence distributions were compared between the two groups using Gray’s k-sample test while treating death without the event of interest as a competing risk event. Multivariable cox proportional hazards regression model was used to calculate hazard ratios with 95% confidence intervals for overall mortality. Multivariable Fine-Gray sub-distribution hazard model was used to calculate hazard rations with 95% confidence interval for bleeding outcome while treating death without bleeding as competing risk event. A p-value <0.05 was considered statistically significant. The total number of variables included in the multivariate models was limited by the total number of outcome events. Several variables were grouped to ensure inclusion while maintaining statistical feasibility of the model. Variables included in univariate analysis were chosen based on available data and clinical significance shown in prior literature and clinical interest based on the study question. Multivariate analysis included statistically significant and clinically relevant variables from the univariate analysis.

The time in therapeutic range (TTR) was calculated for patients on warfarin using all available INR values during the study period. The TTR is defined as the percentage of time the patients’ INRs were within therapeutic range (INR 2–3) using Rosendaal’s method [13]. Rosendaal’s method was also used to calculate the time in supratherapeutic range (INR >3). All analyses were performed using SAS Version 9.4 (SAS Institute Inc, Cary, NC).

## Results

### Baseline characteristics

**Fig 1:**
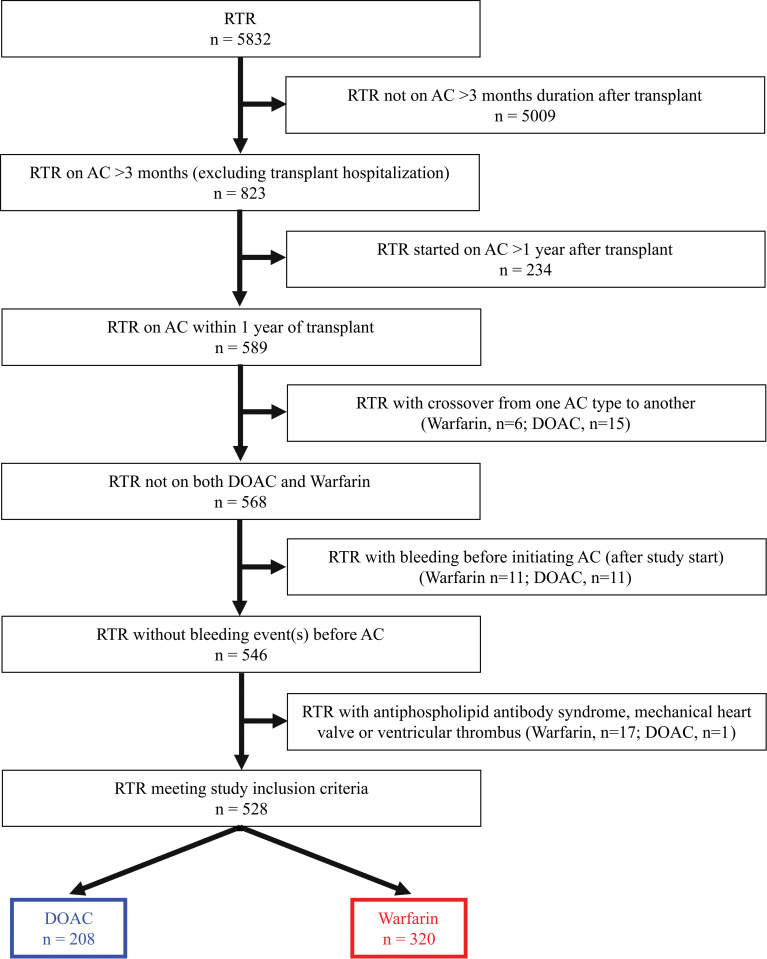
Study population selection. Fig 1 shows the inclusion and exclusion criteria for the study participant selection.

The study population included 208 RTR on DOAC (apixaban 90.9%, rivaroxaban 9.1%) and 320 RTR on warfarin (Fig 1). There were 63 patients in the DOAC group (30.3%) and 111 patients in the warfarin group (34.7%) that were on anticoagulation before transplant, continuing post-transplant for a prolonged period (greater than 3 months) and the remainder of the patients initiated anticoagulation post-transplant (S1 Fig). Warfarin was the anticoagulant type used most frequently for patients initiating anticoagulation at the beginning of the study period, but DOAC use increased in an upward trend during the course of the study and was more frequently employed for patients initiating anticoagulation after 2016 (S1 Table). The median follow-up was 796 days (IQR 371, 1261). There were no significant differences in baseline (time of transplant) age, gender, BMI, diabetes, cardiovascular disease, atrial fibrillation, VTE, GI bleeding, ICH, or ischemic stroke, between those on DOACs vs. warfarin (Table 1). Past or current smoking and was higher in the DOAC group. There was no difference in post-transplant use of aspirin, other antiplatelet, fluconazole, voriconazole, posaconazole, amiodarone, tacrolimus, cyclosporine, or other immunosuppressant. The use of itraconazole was higher in the DOAC group, although very low counts.

**Table 1:** Characteristics of renal transplant recipients on anticoagulation.

Variable	DOAC (N = 208)	Warfarin (N = 320)	P-value
**Age**, mean years (SD)	58.8 (12.5)	57.5 (13.8)	0.48^1^
**Gender**, n female (%)	66 (31.7%)	121 (37.9%)	0.15^2^
**BMI**, mean kg/m^2^ (SD)	29.2 (5.5)	29.4 (5.4)	0.42^1^
**Follow-up**, median days post-transplant (IQR)	449.0 (338.0, 942.0)	1098.0 (521.0, 1517.0)	<0.0001^1^
**Diabetes Mellitus**, n (%)	52 (25.0%)	63 (19.7%)	0.15^2^
**CAD**, n (%)	20 (9.6%)	30 (9.4%)	0.93^2^
**Hypertension**, n (%)	71 (34.1%)	90 (28.1%)	0.14^2^
**PAD**, n (%)	8 (3.8%)	13 (4.1%)	0.90^2^
**CHF**, n (%)	15 (7.2%)	28 (8.8%)	0.53^2^
**Atrial fibrillation**, n (%)	26 (12.5%)	30 (9.4%)	0.25^2^
**VTE**, n (%)	16 (7.7%)	13 (4.1%)	0.07^2^
**GI bleed**, n (%)	7 (3.4%)	4 (1.3%)	0.10^2^
**ICH**, n (%)	0 (0%)	0 (0%)	
**Ischemic stroke**, n (%)	4 (1.9%)	4 (1.3%)	0.54^2^
**Past or current smoker**, n (%)	46 (22.1%)	48 (15.2%)	0.04^2^
**Aspirin**, n (%)	110 (52.9%)	181 (56.6%)	0.41^2^
**Clopidogrel**, n (%)	18 (8.7%)	31 (9.7%)	0.69^2^
**Prasugrel**, n (%)	0 (0.0%)	1 (0.3%)	0.42^2^
**Ticagrelor**, n (%)	2 (1.0%)	3 (0.9%)	0.98^2^
**Amiodarone**, n (%)	23 (11.1%)	54 (16.9%)	0.06^2^
**Fluconazole**, n (%)	117 (56.3%)	177 (55.3%)	0.83^2^
**Itraconazole**, n (%)	7 (3.4%)	1 (0.3%)	0.01^2^
**Voriconazole**, n (%)	4 (1.9%)	12 (3.8%)	0.23^2^
**Posaconazole**, n (%)	5 (2.4%)	10 (3.1%)	0.63^2^
**Diltiazem**, n (%)	26 (12.5%)	42 (13.1%)	0.83^2^
**Tacrolimus**, n (%)	198 (95.2%)	306 (95.6%)	0.82^2^
**Cyclosporine**, n (%)	12 (5.8%)	11 (3.4%)	0.20^2^
**Neither CNI**, n (%)	7 (3.4%)	12 (3.8%)	0.82^2^

^1^Kruskal-Wallis p-value

^2^ Chi-Square p-value

Age, gender and medical diagnosis are defined at baseline (time of renal transplant). Medications are noted for any time during the study period post-transplant.

Body mass index (BMI), Coronary artery disease (CAD), congestive heart failure (CHF), calcineurin inhibitor (CNI), gastrointestinal (GI), intracerebral hemorrhage (ICH), peripheral arterial disease (PAD), venous thromboembolism (VTE)

### Primary and secondary outcomes

**Fig 2:**
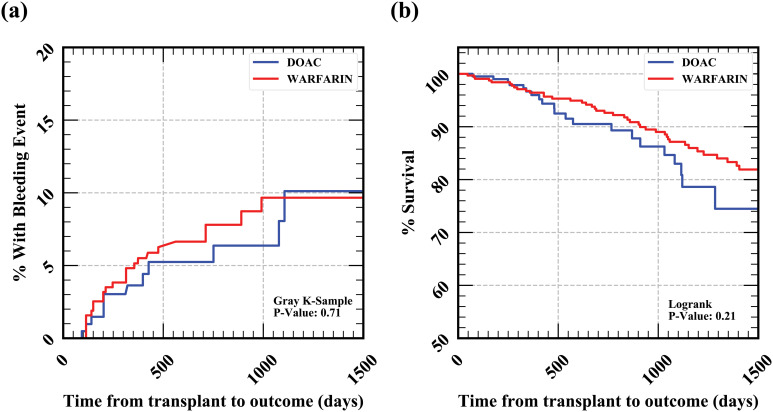
a) Bleeding and b) Mortality Outcomes for DOAC compared to Warfarin in RTR. Fig 2a shows the percent with bleeding events after transplant for RTR anticoagulated with DOACs vs. warfarin. Fig 2b shows the mortality after transplant for RTR on DOACs vs. warfarin.

Overall major bleeding (47 events) following initiation of anticoagulation was higher in the warfarin group compared to the DOAC group (10.6% vs 6.3%, p = 0.71), although the p-value didn’t meet criteria for significance. When the type of bleeding was broken down, the warfarin group had non-significant trend towards increased incident major bleeding (8.4 vs. 5.3%, p = 0.89), GI bleeding (4.4% vs. 1.9%, p = 0.98), ICH (1.9% vs. 1.4%, p = 0.85) compared to the DOAC group (Table 2). The cumulative bleeding incidence at one year, two years, and three years post-transplant was 3.6%, 5.2%, and 8.1% for DOACs compared to 5.1%, 7.8% and 9.7% for warfarin. Bleeding stratified by anticoagulant group over time is shown in Fig 2. No variables, including DOAC vs. warfarin, were significant predictors of bleeding in multivariate analysis (S4 Table).

**Table 2:** Incident bleeding, mortality, and secondary outcomes for renal transplant recipients on anticoagulation.

Outcome	NOAC (N = 208)	Warfarin (N = 320)	P-value^1^
**Major Bleeding**, n (%)	11 (5.3%)	27 (8.4%)	0.89
**GI Bleeding**, n (%)	4 (1.9%)	14 (4.4%)	0.98
**ICH**, n (%)	3 (1.4%)	6 (1.9%)	0.85
**Mortality**, n (%)	21 (10.1%)	71 (22.2%)	0.21
**VTE**, n (%)	74 (35.6%)	125 (39.1%)	0.65
**Ischemic stroke**, n (%)	11 (5.3%)	15 (4.7%)	0.27
**Renal Graft Failure**, n (%)	23 (11.1%)	119 (37.2%)	<0.0001

^1^Log-rank test was used for mortality and Gray’s k-sample test was used for other outcomes while treating death as competing risk event.

Gastrointestinal (GI), intracerebral hemorrhage (ICH), venous thromboembolism (VTE)

Mortality was not significantly different between the warfarin group and the DOAC group when accounting for the longer follow-up time with warfarin (time to event analysis) (22.2% vs. 10.1%, p = 0.21; Table 2). The overall survival at one year, two years, and three years post-transplant was 96.7%, 90.5%, and 83.0% for DOACs compared to 96.4%, 93% and 87.1% for warfarin. Mortality stratified by anticoagulant group over time is shown in Fig 2. Only older age and diabetes were significant predictors of mortality in multivariate analysis. Anticoagulant group was not predictive (S5 Table).

The differences between groups for the primary outcomes were explained by the longer follow-up time for the warfarin group compared to the DOAC group. The median follow-up was significantly longer for the warfarin group (1098 days IQR [521, 1517]) than for the DOAC group (449 days IQR [338, 942]). Fig 2 also demonstrates this for both outcomes (nonsignificant p-values).

VTE, atrial fibrillation and stroke post-transplant were not significantly different between DOAC and warfarin groups (Table 2). Renal graft failure was significantly higher in those on warfarin compared to DOAC (37.2% vs. 11.1%, p<0.0001) and this relationship remained significant when accounting for the varied durations of follow-up (S2 Fig). The incident renal graft failure at one year, two years, and three years post-transplant was 10.3%, 11.8%, and 11.8% for DOACs compared to 33.5%, 36.7% and 37.7% for warfarin.

The mean and median TTR excluding any INR within 6 week of initiation was 35.9% (SD 29.8%) and 36.6% (IQR 4.0% to 60.5%), respectively. The mean and median time in supratherapeutic range was 9.6% (SD 13.6%) and 2.5% (IQR 0% to 78.8%), respectively.

### DOAC dosing analysis

Sixty-seven patients (32%) on DOACs were dose reduced. There were no significant differences in baseline characteristics or comorbidities between the dose-reduced and standard DOAC groups (S6 Table). Those in the dose-reduced group were more likely to be on amiodarone (25.4% vs. 3.5%, p < 0.0001) of assessed interacting medications. They were more likely to be on itraconazole (7.5% vs. 1.4%, p = 0.02), although the absolute number on itraconazole was low (n = 7). and Posaconazole and voriconazole use were non-significantly higher in the dose-reduced group. 51% of those dose reductions were warranted based on assessed factors.

Incident bleeding was not significantly different between those on dose-reduced and standard DOAC although the separation of the Kaplan-Meier curves showed a trend towards favorable bleeding outcomes in the standard dose DOAC group (S7 Table and S3A Fig). Specifically, ICH, GI bleeding and major bleeding were all non-significantly higher in the dose-reduced group (1.5% vs. 1.4%, p = 0.98; 4.5% vs. 0.7%, p = 0.05; and 7.5% vs. 4.3%, p = 0.20, respectively). The absolute event counts were low. The absolute event counts were low. Mortality was not significantly different between those on dose-reduced and standard DOAC although death appeared more infrequent in the dose-reduced group (6.0% vs. 12.1%, p = 0.18) (S7 Table and S3B Fig). There were no significant differences in incident VTE, ischemic stroke or renal graft failure between the dose-reduced and standard DOAC groups (28.4% vs. 39.0%, p = 0.11; 3.0% vs. 6.4%, p = 0.31; and 16.4% vs. 7.8%, p = 0.06, respectively). There appeared to be a high number of VTE events following initiation of anticoagulation. 7% of patients that were not dose reduced should have been based on assessed parameters.

## Discussion

Renal transplant patients represent a relatively understudied group regarding the risks and benefits of DOACs [11]. To our knowledge, this is the largest study to date that compares DOACs to warfarin in RTR. Our study shows similar safety as well as efficacy outcomes compared to warfarin. There was some superiority in primary outcomes that was accounted for by longer follow-up for the warfarin group (non-significant in time-to-event analysis). Existing literature regarding DOAC use in RTR involves small populations, often with low bleeding events limiting conclusions on safety [11, 14–17]. Other studies pool multiple transplant types, limiting interpretation of results in RTR [15, 18, 19].

A recent study found no significant difference in major bleeding in RTR on DOAC (n = 98) compared to warfarin although events were fewer in the DOAC group (7.2% vs. 11.4% per year, p = 0.15). There was no difference in composite bleeding or VTE between groups either. Patients were initiated on anticoagulation late after transplant (median 6.5 years) with median follow-up 12.3 months. Another smaller recent study found that compared to warfarin, DOACs in RTR (n = 52) were associated with a lower bleeding rate (HR 0.39, p = 0.04), although this referred to minor bleeding. This study had a short follow-up (mean 14.1 ± 13 months) and few outcome events (no major bleeding events, stroke, VTE, death or graft loss in the DOAC group). Inclusion criteria required only 1 month of anticoagulation, and the DOAC was started late after transplantation (mean 7.3 ± 7.9 years). Other studies are smaller (n = 42, 23, 31) [11, 16, 17] and had minimal bleeding events to draw conclusions (<5) or assessed RTR late after transplant (e.g., mean time of anticoagulation initiation 8.8 ± 7 years post-transplant) [11]. These studies also appeared supportive of safety of DOACs in RTR. Pooling solid organ transplant data facilitates larger populations to assess significance. A recent meta-analysis of DOAC (n = 259) compared to warfarin use in solid organ transplants noted significantly improved composite bleeding outcomes and no difference in major bleeding [19].

Our relatively large study aligns with the literature showing no increased bleeding outcomes with DOACs vs. warfarin, with possible additional outcome benefits. No other variables studied were influential regarding bleeding or mortality outcomes (significance or magnitude) in multivariate analysis. The protective effect seen by smoking is likely inaccurate, presumably related to survivorship bias or selection bias (i.e. the sample of “healthier” smokers or former smokers chosen for renal transplant is not indicative of the average smoking population). As in the aforementioned studies, the majority of DOACs employed were apixaban followed by rivaroxaban. Some of the other studies had a handful of patients on dabigatran, but none were on this DOAC in our study. Importantly, our study was limited to those initiating anticoagulation within the first year post-transplant or continuing anticoagulation pre- to post-transplant. Many of the other studies evaluated patients initiating anticoagulation many years post-transplant, which does not capture the dynamics seen post-transplant when patients have fluctuating renal function, more interacting anti-rejection and infection prophylaxis medications, and protocol kidney biopsies. This choice of timeframe likely explains why dabigatran use was not seen in our population compared to the other literature: dabigatran is more renally excreted than the aforementioned anti-Xa inhibitors and thus a riskier choice before graft stability is achieved.

Our study showed a high number of RTRs on dose-reduced apixaban. This is not unexpected given the recent post-transplant timeframe chosen, which entails more interacting medications and renal dysfunction compared to remote post-transplant. Drug-drug interactions between cytochrome P-450 (CYP) and P-glycoprotein (P-gp) inhibitors and DOACS (substrates of CYP3A4 and P-gp) can lead to adverse drug events (may increase DOAC serum concentrations and lead to bleeding) [20, 21]. Common such interacting medications in RTRs include CNIs, antifungals, and antiarrhythmics (e.g., amiodarone, diltiazem), which were assessed in this study. Despite mechanistic concerns, studies have not noted significant interactions of apixaban or rivaroxaban with CNIs, including no increased bleeding episodes [17]. Closer CNI monitoring may be warranted with concomitant use of DOAC to monitor for drug-drug interactions, but this is beyond the scope of the present study. Not unexpectedly, the dose-reduced DOAC patients in our study had a higher prevalence of many of the assessed interacting medications, particularly those antifungals which are strong and/or combination inhibitors. It is not unexpected that there was a trend towards higher bleeding in the dose-reduced DOAC group as these patients were likely selected for dose reduction based on their increased bleeding risk based on variables assessed or not assessed in this study.

There was a fairly high percentage RTRs treated with inappropriately dose-reduced apixaban and a small percentage that were improperly treated with standard-dosed apixaban. The assessment of appropriateness (described in the supporting information) is according to packaging and guidelines for dose reduction, which may not be conservative enough in this unique population. We assessed major drug interactions and available graft function data, but this likely did not provide a comprehensive clinical assessment of our study participants on DOACs. The dosing decisions for these patients would have been undertaken in the real-life setting. Our institution’s practice for RTR in the early post-transplant setting is to manage care judiciously with a multidisciplinary team including a transplant pharmacist. It is notable that bleeding outcomes, mortality, and efficacy outcomes (incident VTE and stroke) were not significantly different between groups, which may support appropriate selection. Graft failure was likely higher in the dose-reduced group as these patients had more renal dysfunction necessitating the dose adjustment.

There was a higher use warfarin in our study compared to DOAC for anticoagulation in RTR (60%). There was increasing use of DOACs for initiation of anticoagulation as the study progressed, with preference noted for DOACs after 2016. We hypothesize this is due to increasing familiarity with these agents since their introduction. Warfarin continued to be used frequently, however. Clinicians needing to start anticoagulation in this population for any indication may be more hesitant to utilize DOACs due to fluctuating renal function post-transplant (given uncertainty regarding proper dosing in renal dysfunction and lack of reliable monitoring methods) and less research with these agents in the RTR population. Drug interactions may also be of concern [5–8]. There is greater experience with warfarin use in RTR, although it is also relatively understudied in this distinct population and concerns over efficacy and mortality benefit have been raised [22].

RTRs may be less likely to receive anticoagulation than the general population [22]. This could be related to the aforementioned complicating factors of drug interactions, fluctuating renal function, as well as the need for procedures or biopsies in RTRs. Literature also shows increased bleeding in solid organ transplant recipients on anticoagulation for VTE compared to non-recipients [23]. The bleeding rates in our study are higher than in the general population, but fairly consistent with previous literature although the range is quite varied likely due to differences in follow-up, duration of anticoagulation and timing of anticoagulation post-transplant [15]. Anticoagulant related nephropathy, a newly recognized form of acute kidney injury secondary to anticoagulation, may be of greater concern in this population with a propensity for kidney injury [24]. Furthermore, there may be some lingering hesitancy using anticoagulation in these patients prior to transplant. This relates to concerns regarding the overall benefit (insufficient reduction in thromboembolic events) of anticoagulation in ESRD patients with atrial fibrillation [25, 26], conflicting or unclear guidelines [10, 27, 28], and increased bleeding risks compared to the general population [29, 30]. The major cause of stroke among patients with ESRD is of vascular origin, which may explain the lack of benefit seen with anticoagulation. However, no reduction in stroke with aspirin or clopidogrel alone compared to non-use in ESRD patients was seen in some studies [31–33]. It is not fully clear how the pathophysiology of incident stroke changes post renal transplant and what risk remains from ESRD cause, raising the question of what factors may attenuate beneficial effects of anticoagulation for atrial fibrillation in RTR compared to the general population.

Renal graft failure was less in the DOAC group compared to the warfarin group. However, we did not complete a comprehensive assessment of relevant variables including graft type to draw conclusions. The relationship between anticoagulant type and graft failure is not well studied. Most of the aforementioned small studies comparing DOAC to warfarin did not assess graft loss or had no graft loss events. Graft thrombosis represents a main cause of graft failure in the first year [34]. The median time to graft failure in our study was 59 days (IQR 33, 149), which was similar between both anticoagulant groups (60 days for patients on DOAC and 59 days for those on warfarin). Potentially, DOACs may be more effective than warfarin in combatting this type of thrombosis, although this is unknown. Further study is needed to adequately assess variables associated with graft failure in this cohort of patients to determine the contribution (if any) of anticoagulant type to the risk for graft failure.

### Limitations

The study has the inherent limitations of a retrospective study design. Namely, anticoagulant choice was at the treating physician’s discretion. Accounting for numerous confounding factors cannot fully account for complex clinical circumstances that lead to decisions for one anticoagulant type or dosing. The indication for anticoagulation was not assessed although we chose specific inclusion and exclusion criteria to limit the population to patients on anticoagulation for atrial fibrillation or VTE. Specifically, we included those on anticoagulation longer than 3 months which would largely exclude those on anticoagulation for DVT with reversible risk factors and those with superficial venous thrombosis. We also excluded those with mechanical heart valves, antiphospholipid antibody syndrome and ventricular thrombus. Dose reduction of anticoagulation for VTE after the initial treatment period was not practiced at our institution during the study period. Bleeding risk assessment (i.e. HAS-BLED) as well as the CHA2DS2-vasc assessment were not collected due to difficulties associated with manual data pull for this large study.

The low TTR seen for participants on warfarin is not unexpected given there was little INR data available as presumably routine INR monitoring for assessed patients is done outside of the Mayo system. Only 57% of patients had INR data and those that did had few values. Furthermore, many patients likely had INR checks within the Mayo System during situations that would necessitate an INR check deviating from their normal steady-state. Similarly, because patients were captured beyond 1 month after transplant and because the majority of RTR recipients are transitioned back to their referring nephrologist after 4 months, there was insufficient data on creatinine and CNI levels that were easily accessible from the Mayo medical records. Lastly, the study did not account for other known variables associated with graft failure. As such, further studies are needed to assess if there is a difference in graft failure associated with the type of anticoagulation.

## Conclusions

Our study found that DOACs did not have inferior bleeding or mortality outcomes compared to warfarin in RTRs on anticoagulation for more than 3 months within a year after kidney transplant. Secondary efficacy outcomes were also non-inferior. Renal graft failure was less in the DOAC group. The findings of our study align with the current literature showing non-inferiority of DOACs compared to warfarin regarding safety, with a possible favorable risk profile. Further studies with longer follow-up are needed to define benefit of DOACs compared to warfarin in RTRs. There was a high rate of improper DOAC dose reduction in our study according to standard guidelines, but such guidelines may not be adequate for this distinct patient population. The dose-reduced DOAC group had non-inferior outcomes compared to the standard-dosed group. The decision for dose reduction in RTRs is challenging and ideally involves a multidisciplinary team including a pharmacist. Our study of DOAC use in RTR in clinical practice indicates that DOACs appear to be as safe as warfarin.

## Supporting information

S1 Fig Anticoagulant use by type and timing of initiation. The timing of initiation of DOAC and warfarin relative to transplant is shown.
(EPS)

S2 Fig Renal graft failure for DOAC compared to warfarin in RTR. S2 Fig shows the percent with renal graft failure for RTR anticoagulated with DOACs vs. warfarin.
(EPS)

S3 Fig a. Bleeding and b. Mortality Outcomes for Standard Compared to Dose-Reduced DOAC in RTR. S3a Fig shows the percent with bleeding events after transplant for RTR anticoagulated with standard vs. dose-reduced DOAC. S3b Fig shows the mortality after transplant for RTR on standard vs. dose-reduced DOAC.
(EPS)

S1 Table Anticoagulation type initiated annually. (DOCX)

S2 Table Univariate analysis for bleeding in renal transplant recipients on prolonged anticoagulation. (DOCX)

S3 Table Univariate analysis for mortality in renal transplant recipients on prolonged anticoagulation. (DOCX)

S4 Table Multivariate analysis for bleeding in renal transplant recipients on prolonged anticoagulation. (DOCX)

S5 Table Multivariate analysis for mortality in renal transplant recipients on prolonged anticoagulation. (DOCX)

S6 Table Characteristics of renal transplant recipients on standard dose and dose-reduced DOAC. (DOCX)

S7 Table Incident bleeding, mortality, and secondary outcomes for renal transplant recipients on standard and dose-reduced DOAC. (DOCX)

S8 Table ICD diagnosis codes used to define variables and outcomes. (DOCX)

S1 Text Outcome verification method. (DOCX)

S2 Text DOAC dose reduction assessment criteria. (DOCX)

S1 Dataset De-identified data used for analysis. (CSV)
