# Supplementary material for: Safety and mortality outcomes for direct oral anticoagulants in renal transplant recipients
Source: PLoS One. 2023 May 16;18(5):e0285412. doi: 10.1371/journal.pone.0285412 (PMC10187891; doi:10.1371/journal.pone.0285412)
Supplement: S1 Table — (DOCX) [file pone.0285412.s004.docx]

**S1 Table. Anticoagulation Type Initiated Annually.**

|  | **DOAC** | **Warfarin** | **Total** | **P-value** |
| --- | --- | --- | --- | --- |
|  | (N=208) | (N=320) | (N=528) |  |
| **Year of Anticoagulation Initiation, n (%)** |  |  |  | <0.001^1^ |
| 2011 | 0 (0.0%) | 18 (5.6%) | 18 (3.4%) |  |
| 2012 | 0 (0.0%) | 21 (6.6%) | 21 (4.0%) |  |
| 2013 | 0 (0.0%) | 17 (5.3%) | 17 (3.2%) |  |
| 2014 | 3 (1.4%) | 36 (11.3%) | 39 (7.4%) |  |
| 2015 | 4 (1.9%) | 58 (18.1%) | 62 (11.7%) |  |
| 2016 | 18 (8.7%) | 56 (17.5%) | 74 (14.0%) |  |
| 2017 | 24 (11.5%) | 35 (10.9%) | 59 (11.2%) |  |
| 2018 | 40 (19.2%) | 29 (9.1%) | 69 (13.1%) |  |
| 2019 | 20 (9.6%) | 19 (5.9%) | 39 (7.4%) |  |
| 2020 | 71 (34.1%) | 20 (6.3%) | 91 (17.2%) |  |
| 2021 | 28 (13.5%) | 11 (3.4%) | 39 (7.4%) |  |

^1^Chi-Square p-value
