# Supplementary material for: Safety and mortality outcomes for direct oral anticoagulants in renal transplant recipients
Source: PLoS One. 2023 May 16;18(5):e0285412. doi: 10.1371/journal.pone.0285412 (PMC10187891; doi:10.1371/journal.pone.0285412)
Supplement: S2 Table — (DOCX) [file pone.0285412.s005.docx]

**S2 Table. Univariate Analysis for Bleeding in Renal Transplant Recipients on Prolonged Anticoagulation.**

| **Variable** | **HR (95% CI)** | **p-value** |
| --- | --- | --- |
| DOAC (vs. Warfarin) | 1.37 (0.88, 2.15) | 0.17 |
| Age (one year increase) | 1.02 (1.00, 1.04) | 0.02 |
| Aspirin | 0.96 (0.63, 1.48) | 0.86 |
| Other Antiplatelet | 1.00 (0.50, 2.00) | 1.00 |
| History of Stroke | 0.94 (0.13, 6.76) | 0.95 |
| 6 week Creatinine  (per 1.0mg/dL increase) | 1.27 (1.13, 1.43) | <0.001 |

Death without bleeding treated as competing risk event.

Prior bleeding not included as frequency very rare.
