# Supplementary material for: Safety and mortality outcomes for direct oral anticoagulants in renal transplant recipients
Source: PLoS One. 2023 May 16;18(5):e0285412. doi: 10.1371/journal.pone.0285412 (PMC10187891; doi:10.1371/journal.pone.0285412)
Supplement: S3 Table — (DOCX) [file pone.0285412.s006.docx]

**S3 Table. Univariate Analysis for Mortality in Renal Transplant Recipients on Prolonged Anticoagulation.**

| **Variable** | **HR (95% CI)** | **p-value** |
| --- | --- | --- |
| DOAC (vs. Warfarin) | 1.41 (0.82, 2.40) | 0.21 |
| Age (one year increase) | 1.06 (1.03, 1.09) | <0.001 |
| Female Gender (vs. Male) | 1.23 (0.74, 2.05) | 0.42 |
| Diabetes Mellitus | 2.33 (1.39, 3.91) | <0.001 |
| Cardiac Disease | 1.06 (0.54, 2.10) | 0.86 |
| Vascular Disease | 1.45 (0.53, 4.00) | 0.47 |
| Active or Former Smoker | 0.30 (0.11, 0.84) | 0.02 |
| Atrial Fibrillation | 1.65 (0.81, 3.34) | 0.16 |
| Aspirin | 1.23 (0.73, 2.08) | 0.43 |
| 6 week Creatinine  (per 1.0 mg/dL increase) | 1.31 (1.15, 1.50) | <0.001 |

Cardiac disease includes coronary artery disease and congestive heart failure. Vascular disease includes peripheral arterial disease and ischemic stroke.

Missing creatinine data: n=18 for NOAC, n =19 for warfarin
