# Supplementary material for: Safety and mortality outcomes for direct oral anticoagulants in renal transplant recipients
Source: PLoS One. 2023 May 16;18(5):e0285412. doi: 10.1371/journal.pone.0285412 (PMC10187891; doi:10.1371/journal.pone.0285412)
Supplement: S4 Table — (DOCX) [file pone.0285412.s007.docx]

**S4 Table. Multivariate Analysis for Bleeding in Renal Transplant Recipients on Prolonged Anticoagulation**

| **Variable** | **HR (95% CI)** | **p-value** |
| --- | --- | --- |
| DOAC (vs. warfarin) | 0.87 (0.43, 1.74) | 0.69 |
| Age (one year increase) | 0.99 (0.97, 1.01) | 0.45 |
| Aspirin | 0.81 (0.41, 1.59) | 0.54 |
| Other Antiplatelet | 0.69 (0.18, 2.62) | 0.59 |
| 6-week creatinine (per 1.0mg/dL increase) | 2.55 (0.26, 25.52) | 0.43 |

Death without bleeding treated as competing risk event.

Prior bleeding not included as frequency very rare.
