# Supplementary material for: Safety and mortality outcomes for direct oral anticoagulants in renal transplant recipients
Source: PLoS One. 2023 May 16;18(5):e0285412. doi: 10.1371/journal.pone.0285412 (PMC10187891; doi:10.1371/journal.pone.0285412)
Supplement: S5 Table — (DOCX) [file pone.0285412.s008.docx]

**S5 Table.** **Multivariate Analysis for Mortality in Renal Transplant Recipients on Prolonged Anticoagulation.**

| **Variable** | **HR (95% CI)** | **p-value** |
| --- | --- | --- |
| DOAC (vs. warfarin) | 1.64 (0.93, 2.89) | 0.09 |
| Age (one year increase) | 1.06 (1.03, 1.09) | <0.001 |
| Diabetes Mellitus | 1.73 (1.02, 2.95) | 0.04 |
| Active or Former Smoker | 0.33 (0.12, 0.92) | 0.03 |
| 6 week Creatinine  (per 1.0 mg/dL increase) | 0.33 (0.12, 0.92) | 0.03 |

Cardiac disease includes coronary artery disease and congestive heart failure. Vascular disease includes ischemic stroke and peripheral arterial disease.
