# Supplementary material for: Safety and mortality outcomes for direct oral anticoagulants in renal transplant recipients
Source: PLoS One. 2023 May 16;18(5):e0285412. doi: 10.1371/journal.pone.0285412 (PMC10187891; doi:10.1371/journal.pone.0285412)
Supplement: S6 Table — (DOCX) [file pone.0285412.s009.docx]

**S6 Table. Characteristics of Renal Transplant Recipients on Standard Dose and Dose-Reduced DOAC.**

| **Variable** | **Standard DOAC (N=141)** | **Dose-Reduced DOAC (N=67)** | **P-value** |
| --- | --- | --- | --- |
| **Age**, mean years (SD) | 58.7 (11.95) | 58.9 (13.64) | 0.90^1^ |
| **Gender**, n female (%) | 43 (30.5%) | 24 (35.8%) | 0.44^2^ |
| **BMI**, mean kg/m^2^ (SD) | 29.3 (5.53) | 28.8 (5.52) | 0.63^1^ |
| **Follow-up**, median days post-transplant (IQR) | 446.5 (339.5, 908.5) | 451.0 (321.0, 1087.0) | 0.69^1^ |
| **Diabetes Mellitus**, n (%) | 35 (24.8%) | 17 (25.4%) | 0.93^2^ |
| **CAD**, n (%) | 14 (9.9%) | 6 (9.0%) | 0.82^2^ |
| **Hypertension**, n (%) | 48 (34.0%) | 24 (35.8%) | 0.80^2^ |
| **PAD**, n (%) | 5 (3.5%) | 3 (4.5%) | 0.74^2^ |
| **CHF**, n (%) | 9 (6.4%) | 5 (7.5%) | 0.77^2^ |
| **Atrial fibrillation**, n (%) | 15 (10.6%) | 11 (16.4%) | 0.24^2^ |
| **VTE**, n (%) | 10 (7.1%) | 6 (9.0%) | 0.64^2^ |
| **GI bleed**, n (%) | 3 (2.1%) | 4 (6.0%) | 0.15^2^ |
| **ICH**, n (%) | 0 (0%) | 0 (0%) |  |
| **Ischemic stroke**, n (%) | 2 (1.4%) | 2 (3.0%) | 0.44^2^ |
| **Past or current smoker**, n (%) | 27 (19.1%) | 20 (29.9%) | 0.08^2^ |
| **Aspirin**, n (%) | 73 (51.8%) | 37 (55.2%) | 0.64^2^ |
| **Clopidogrel**, n (%) | 11 (7.8%) | 7 (10.4%) | 0.53^2^ |
| **Prasugrel**, n (%) | 0 (0%) | 0 (0%) |  |
| **Ticagrelor**, n (%) | 1 (0.7%) | 1 (1.5%) | 0.59^2^ |
| **Amiodarone**, n (%) | 5 (3.5%) | 17 (25.4%) | <0.0001^2^ |
| **Fluconazole**, n (%) | 84 (59.6%) | 33 (49.3%) | 0.16^2^ |
| **Itraconazole**, n (%) | 2 (1.4%) | 5 (7.5%) | 0.02^2^ |
| **Voriconazole**, n (%) | 1 (0.7%) | 3 (4.5%) | 0.06^2^ |
| **Posaconazole**, n (%) | 2 (1.4%) | 3 (4.5%) | 0.18^2^ |
| **Diltiazem**, n (%) | 18 (12.8%) | 7 (10.4%) | 0.63^2^ |
| **Tacrolimus**, n (%) | 132 (93.6%) | 66 (98.5%) | 0.12^2^ |
| **Cyclosporine**, n (%) | 10 (7.1%) | 3 (4.5%) | 0.47^2^ |
| **Neither CNI**, n (%) | 7 (5.0%) | 0 (0.0%) | 0.06^2^ |
| **Cr 6 weeks**, mean mL/min (SD) | 1.7 (0.9) | 1.8 (0.6) | 0.18^1^ |
| **Cr one year**, mean mL/min (SD) | 1.4 (0.4) | 1.7 (0.6) | 0.008^1^ |
| **Cr last follow-up**, mean mL/min (SD) | 1.6 (1.0) | 1.9 (1.4) | 0.008^1^ |

^1^Kruskal-Wallis p-value

^2^ Chi-Square p-value

Age, gender and medical diagnosis are defined at baseline (time of renal transplant). Medications are noted for any time during the study period post-transplant.

Body mass index (BMI), Coronary artery disease (CAD), congestive heart failure (CHF), calcineurin inhibitor (CNI), gastrointestinal (GI), intracerebral hemorrhage (ICH), peripheral arterial disease (PAD), venous thromboembolism (VTE)
