# Supplementary material for: Safety and mortality outcomes for direct oral anticoagulants in renal transplant recipients
Source: PLoS One. 2023 May 16;18(5):e0285412. doi: 10.1371/journal.pone.0285412 (PMC10187891; doi:10.1371/journal.pone.0285412)
Supplement: S7 Table — (DOCX) [file pone.0285412.s010.docx]

**S7 Table. Incident Bleeding, Mortality, and Secondary Outcomes for Renal Transplant Recipients on Standard and Dose-Reduced DOAC.**

| **Outcome** | **Standard DOAC (N=141)** | **Dose-Reduced DOAC (N=67)** | **P-value**^1^ |
| --- | --- | --- | --- |
| **Major Bleeding**, n (%) | 6 (4.3%) | 5 (7.5%) | 0.20 |
| **GI Bleeding**, n (%) | 1 (0.7%) | 3 (4.5%) | 0.05 |
| **ICH**, n (%) | 2 (1.4%) | 1 (1.5%) | 0.98 |
| **Mortality**, n (%) | 17 (12.1%) | 4 (6.0%) | 0.18 |
| **VTE**, n (%) | 55 (39.0%) | 19 (28.4%) | 0.11 |
| **Ischemic stroke**, n (%) | 9 (6.4%) | 2 (3.0%) | 0.31 |
| **Renal Graft Failure**, n (%) | 11 (7.8%) | 11 (16.4%) | 0.06 |

^1^Log-rank test was used for mortality and Gray’s k-sample test was used for other outcomes while treating death as competing risk event.

Gastrointestinal (GI), intracerebral hemorrhage (ICH), venous thromboembolism (VTE)
