# Supplementary material for: Safety and mortality outcomes for direct oral anticoagulants in renal transplant recipients
Source: PLoS One. 2023 May 16;18(5):e0285412. doi: 10.1371/journal.pone.0285412 (PMC10187891; doi:10.1371/journal.pone.0285412)
Supplement: S8 Table — (DOCX) [file pone.0285412.s011.docx]

**S8 Table. ICD Diagnosis Codes Used to Define Variables and Outcomes.**

| **Diagnosis or Outcome** | **ICD Codes** |
| --- | --- |
| Diabetes Mellitus | E08, E09, E10, E11, E13, 249, 250, 357.2, 362, 366.41 |
| Atrial fibrillation | I48, 427.3 |
| Hypertension | I10, 401 |
| Congestive Heart failure | I11, I13, N18, I50, 402.11, 402.91, 404, 428, |
| Coronary artery disease | I25, 412, 414, 429.2 |
| Peripheral Arterial disease | I70, I73.9, 440, 443 |
| Major Bleeding | H05.2, H43, H44, I23, I31.2, I60, I61, I62, J94.2, K22.6, K25.0, K26.0, K26.2, K26.4, K26.6, K27.0, K27.2, K27.4, K27.6, K28.0, K28.2, K28.4, K28.6, K31.82, K55.21, K62.5, K66.1, K92.2, M25.0, R04, R58, S06.4X, S06.5X, S06.6X, S26.0, S27.1, 360.43, 376.32, 379.23, 423.0, 429.79, 430, 431, 432, 459.0, 511.89, 530.7 530.82, 531, 532, 533, 534, 535, 537, 562, 568.81, 569.3, 569.85, 578.9, 596.7, 719.1-19, 784.8, 786.39, 800.21-25, 800.72-4, 801.7, 803.2, 803.7, 804.2, 852, 853 |
| GI Bleeding | I85.01, I 85.11, K22.11, K22.6, K25, K26, K27, K28, K29, K31.8, K55.21, K57, K62.5, K66.1, K92, 456, 530, 531, 532, 533, 534, 535, 537.83, 537.84, 562, 569.3, 569.85, 578, |
| Intracranial Hemorrhage | I60, I61, I62, S06.4X, S06.5X, S06.6X, 430, 431, 432, 800, 801, 803, 804, 852, 853 |
| Venous thromboembolism | I26, I82, 415, 453 |
| Ischemic Stroke | I63, 433, 434 |
| Renal graft failure | T86.12, 996.81 |

Full list of codes is available upon request.
