# Supplementary material for: Safety and mortality outcomes for direct oral anticoagulants in renal transplant recipients
Source: PLoS One. 2023 May 16;18(5):e0285412. doi: 10.1371/journal.pone.0285412 (PMC10187891; doi:10.1371/journal.pone.0285412)
Supplement: S1 Text — (DOCX) [file pone.0285412.s012.docx]

**S1 Text. Outcome Verification Method.**

To evaluate the accuracy of automated endpoint collection from the EMR, manual endpoint verification was performed. Endpoints were manually verified through review of provider notes and applicable diagnostic/radiology reports. Major bleeding events defined by ICD codes had high accuracy (91.5%).
