# Supplementary material for: Safety and mortality outcomes for direct oral anticoagulants in renal transplant recipients
Source: PLoS One. 2023 May 16;18(5):e0285412. doi: 10.1371/journal.pone.0285412 (PMC10187891; doi:10.1371/journal.pone.0285412)
Supplement: S2 Text — (DOCX) [file pone.0285412.s013.docx]

**S2 Text. DOAC Dose Reduction Assessment Criteria.**

Apixaban may be dose reduced for the indication of atrial fibrillation if the patient meets 2 of 3 criteria: creatinine (Cr) ≥ 1.5 mg/dL, age ≥ 80 years, or weight ≤ 60 kg; or the Cr clearance (CrCl) is 15-29 mL/min. For any indication, apixaban may be dose reduced if the patient was on a strong cytochrome (CYP) 3A4 and P-glycoprotein (P-gp) inhibitor. According to common practice at our institution, apixaban may be dose reduced if the patient had CrCl <40 mL/min and was on one or more moderate inhibitors. Rivaroxaban may be decreased for the indication of atrial fibrillation for CrCl 15-50 mL/min. The Cr values were assessed at the time closest to 6 weeks.
